# *Malva sylvestris* Inhibits Inflammatory Response in Oral Human Cells. An *In Vitro* Infection Model

**DOI:** 10.1371/journal.pone.0140331

**Published:** 2015-10-19

**Authors:** Bruna Benso, Pedro Luiz Rosalen, Severino Matias Alencar, Ramiro Mendonça Murata

**Affiliations:** 1 Department of Physiological Sciences, Piracicaba Dental School, University of Campinas, Piracicaba, Sao Paulo, Brazil; 2 Department of Agri-food Industry, Food and Nutrition, “Luiz de Queiroz” College of Agriculture, University of Sao Paulo, Piracicaba, Sao Paulo, Brazil; 3 Division of Periodontology, Diagnostic Sciences & Dental Hygiene and Division of Biomedical Sciences Herman Ostrow School of Dentistry, University of Southern California, Los Angeles, United States of America; University of Oklahoma Health Sciences Center, UNITED STATES

## Abstract

The aim of this study was to investigate the *in vitro* anti-inflammatory activity of *Malva sylvestris* extract (MSE) and fractions in a co-culture model of cells infected by *Aggregatibacter actinomycetemcomitans*. In addition, we evaluated the phytochemical content in the extract and fractions of *M*. *sylvestris* and demonstrated that polyphenols were the most frequent group in all samples studied. An *in vitro* dual-chamber model to mimic the periodontal structure was developed using a monolayer of epithelial keratinocytes (OBA-9) and a subepithelial layer of fibroblasts (HGF-1). The invasive periodontopathogen *A*. *actinomycetemcomitans* (D7S-1) was applied to migrate through the cell layers and induce the synthesis of immune factors and cytokines in the host cells. In an attempt to analyze the antimicrobial properties of MSE and fractions, a susceptibility test was carried out. The extract (MIC 175 μg/mL, MBC 500μg/mL) and chloroform fraction (MIC 150 μg/mL, MBC 250 μg/mL) were found to have inhibitory activity. The extract and all fractions were assessed using a cytotoxicity test and results showed that concentrations under 100 μg/mL did not significantly reduce cell viability compared to the control group (p > 0.05, viability > 90%). In order to analyze the inflammatory response, transcriptional factors and cytokines were quantified in the supernatant released from the cells. The chloroform fraction was the most effective in reducing the bacterial colonization (p< 0.05) and controlling inflammatory mediators, and promoted the down-regulation of genes including IL-1beta, IL-6, IL-10, CD14, PTGS, MMP-1 and FOS as well as the reduction of the IL-1beta, IL-6, IL-8 and GM-CSF protein levels (p< 0.05). *Malva sylvestris* and its chloroform fraction minimized the *A*. *actinomycetemcomitans* infection and inflammation processes in oral human cells by a putative pathway that involves important cytokines and receptors. Therefore, this natural product may be considered as a successful dual anti-inflammatory–antimicrobial candidate.

## Introduction

Periodontal disease is characterized by bacterial infection associated with the presence of biofilm, resulting in chronic inflammation of the tooth-supporting tissues and leading to progressive destruction of periodontal tissue. This disease affects up to 90% of the world’s population [1, 2]. Dental biofilm with a large quantity of gram-negative bacteria is responsible for the initiation and maintenance of periodontal inflammation [3]. *Aggregatibacter actinomycetemcomitans* has been described as an important agent of localized aggressive periodontic lesions, but is also related to chronic periodontitis [4, 5]. In addition, *A*. *actinomycetemcomitans* and other pathogenic microbiota including *Porphyromonas gingivalis*, *Treponema denticola*, *Tannerela forsythia* trigger both innate and acquired immune responses, resulting in the progression of periodontal disease, and promote soft tissue inflammation and destruction with consequent bone resorption [6].

The development of new therapeutic agents that can inhibit biofilm formation and modulate the inflammatory response will have a major impact on the prevention and treatment of periodontal disease [7].

Nature has been a source of medicinal products for centuries, yielding many useful drugs [8]. A wide variety of plants are well recognized for their medicinal and nutraceutical value, and the exploration of biodiversity from rich environments has led to the discovery of many pharmacologically active chemicals [9, 10]. *Malva sylvestris* is one example. Commonly known as mallow, it is a plant native to Europe, North Africa and Asia. The ethnopharmacological literature has reported a long history of recognition for its potent anti-inflammatory, antioxidant, anticancer and antiulcerogenic properties [11, 12]. Some reports have indicated that *M*. *sylvestris* contains phytochemicals including several classes of terpenoids, including monoterpenes, diterpenes, sesquiterpenes and norterpenes [11, 13, 14]. Since natural products do not have a standard composition, there is increasing interest in identifying biological therapeutic potential in new plant extracts [15]. Thus, the aim of this study was to investigate *in vitro* the antimicrobial and anti-inflammatory activity of *Malva sylvestris* extract and fractions in a dual chamber model of epithelial and subepithelial cells infected by *A*. *actinomycetemcomitans*.

## Material and Methods

### Preparation of the extract and fractions

*Malva sylvestris* leaves were purchased from a local farmer in the municipality of Princesa Isabel, Paraiba (northeast Brazil) in March and April 2013. This plant is not an endangered or protected species and was registered in the herbarium of the University of Sao Paulo (USP), receiving an identification number (ESA voucher # 121403). Absolute ethanol (800 mL) at room temperature was used to create extracts of *M*. *sylvestris* leaves (100 g) using exhaustive maceration (for 7 days). Filtration was used to obtain the ethanolic extract of *M*. *sylvestris* (MSE). The material was lyophilized, homogenized, weighed and stored at -20°C. The MSE was successively partitioned using liquid-liquid extraction with hexane, chloroform, and ethyl acetate solvents. The final residue obtained after ethyl acetate fractionation was totally soluble in water and thus was called the aqueous fraction (AF)[16]. The extract (MSE), chloroform fraction (CLF) and aqueous fraction were re-suspended in 1% ethanol and used in the biological assays.

#### Determination of total flavonoid, phenol and condensed tannin content

For the flavonoid determination, the aluminum chloride method was used. Total flavonoid contents were calculated using quercetin for the calibration curve. The absorbance was measured at 425 nm with a microplate reader (SpectraMax M5, Molecular Devices Sunnyvale, CA, USA). The polyphenol content was measured by the Folin-Ciocalteu method and gallic acid was used as a standard equivalent [17]. For the content of condensed tannins, a vanillin solution was added to the extract, followed by 37% hydrochloric acid. The calibration curve was determined based on catechin as a reference.

### Bacterial Strains

The *A*. *actinomycetemcomitans* (D7S-1) was cultivated from the subgingival plaque of an African American female patient diagnosed with generalized aggressive periodontitis. The strain was kindly donated by Dr. Casey Chen (University of Southern California) [18]. In addition, the following reference strains were used: *Fusobacterium nucleatum* ATCC 25586, *Prevotella intermedia* 25611 and *Porphyromonas gingivalis* ATCC BAA-308.

### Cell culture

Keratinocytes were processed and isolated, and the cell line established was named OBA-9 [19]. The cell line was kindly donated by Dr. Kusumoto. OBA-9 cells used in this experiment were cultured in a specific medium for keratinocytes (Defined Keratinocyte-SFM, Life Technologies, Carlsbad, CA, USA). The human gingival fibroblasts HGF-1 (ATCC CRL-2014) were cultured in Dulbecco’s modified Eagle’s medium (DMEM) with 10% fetal bovine serum (Gibco, Life Technologies, Carlsbad, CA, USA), 100 U/mL penicillin and 100 μg/mL streptomycin (Invitrogen Life Technologies, CA, USA). Cells were maintained in a humidified incubator at 37°C in 5% CO_2_.

### Susceptibility testing

The susceptibility of four potential periodontopathogenic bacteria (*A*. *actinomycetemcomitans* DS7-1, *Fusobacterium nucleatum* ATCC 25586, *Prevotella intermedia* 25611 and *Porphyromonas gingivalis* ATCC BAA-308) to MSE extract and fractions were tested. Tests were performed according to Clinical and Laboratory Standards Institute guidelines [20]. The minimum inhibitory concentration (MIC) was determined as follows. Bacteria were inoculated at a concentration of 5 × 10^5^ CFU/mL in 96-well microplates, using a trypticase soy broth and yeast extract medium (TSB, YE, Difco, Franklin Lakes, NJ, USA) for *A*. *actinomycetemcomitans* and enriched with 5 μg/mL hemin and 1 μg/mL of menadione for the other microorganisms. The concentrations of MSE and fractions ranged from 3.125 to 1000 μg/mL. The vehicle control was ethanol (final ethanol concentration: 1%, v/v), and the positive control was gentamicin (1 mg/mL, Sigma-Aldrich, St. Louis, MO, USA). The plates for the evaluation of antimicrobial activity against facultative aerobes were incubated at 37°C, 5% CO_2_ and the plates for evaluation of activity against strict anaerobes were placed in an anaerobic chamber at 37°C, 10% H_2_, 10% CO_2_ and 80% N_2_. The MIC was defined as the lowest concentration of MSE or fraction that allowed no visible growth, confirmed by 0.01% resazurin dye (Promega, Madison, WI, USA). The minimum bactericidal concentration (MBC) was determined by subculturing in trypticase soy agar (TSA, Difco, Franklin Lakes, NJ, USA) or TSA containing 2 μg/mL hemin, 1 μg/mL menadione and sheep blood (5.0%) and 20 μL aliquots from each incubated well with a concentration equal to or greater than the MIC. The experiments were conducted in triplicate in three independent assays.

### Cell viability test

HGF-1 cells were seeded (~ 1x10^5^ cells/mL) in a 96-well plate and incubated for 24 h at 37°C with 5% CO_2_. *M*. *sylvestris* extract and fractions (0.1–1000 μg/mL) were added to the cell culture and incubated for 24 h. After the incubation time, the supernatant was discarded and the cells were washed with PBS (Lonza, Walkersville, MD, USA). Fresh medium and 20 μL of CellTiter-Blue (Promega Corp, Madson, WI, USA) were added and incubated at 37°C and 5% CO_2_. The CellTiter-Blue test is a fluorescent assay that measures cell viability via non-specific redox enzyme activity. After incubation, the well contents were transferred to a new microplate and the fluorescence was read in a microplate reader (SpectraMax M5 Molecular Devices Sunnyvale, CA, USA) with 550 nm excitation, 585 nm emission [21].

### Invasion dual chamber assay

The activity of MSE and its fractions in cells infected by *A*. *actinomycetemcomitans* were investigated using an adapted dual chamber model to mimic the periodontum [22]. Keratinocytes (OBA-9) were seeded in a transwell insert with an 8 μm pore and 0.3 cm^2^ culture surface (Grenier Bio-One, Monroe, NC, USA) and positioned in a 24 well plate. The basal chamber was seeded with HGF-1 fibroblasts. After 24 h the transepithelial resistance (TEER) was measured for each cell layer using a Millicell-ERS Volt-Ohm Meter (Millipore, Bedford, MA, USA). The cell layer confluence in the transwell insert was measured to reach the optimal TEER (>150 Ohm/cm^2^). On day 2, an overnight *A*. *actinomycetemcomitans* culture was harvested by centrifugation at 900 X *g* for 10 min at room temperature and incubated in the dual chamber with KSFM culture medium (~1x10^6^ CFU/mL) passing through the upper layer of cells (OBA-9) and reaching the bottom layer (HGF-1) for 2h. Extracellular, unattached bacteria were removed by washing with saline buffer (PBS) two times. After this initial incubation, epithelial and subepithelial cell layers were incubated with gentamicin 100 μg/mL (Sigma, St Louis, MO, USA) to kill the extracellular bacteria. The medium was removed and washed with saline buffer. Fresh new culture medium was added and the culture was treated with MSE or fractions at a concentration of 75 μg/mL. In light of the dose-dependent effects of MSE and CLF treatments, this concentration was determined to be the highest concentration that possessed antimicrobial activity but was still non-cytotoxic after an exposure time of 24 h.

### Sample analysis

#### Antimicrobial activity

The antimicrobial activity of MSE and fractions in the co-culture model was accessed after 24 h of treatment. Aliquots of 20 μL were cultured from each sample in TSB-YE plates to determine the CFU/mL and quantify the numbers of viable bacterial cells.

#### Analysis using the RT^2^ Profiler PCR Array

One microgram of RNA was converted in cDNA using RT^2^ First Strand Kit (Qiagen, Valencia, CA, USA) according to the manufacture’s instructions. 84 genes were analyzed using inflammatory response & Autoimmunity Array RT^2^ profiler (Qiagen Sabiosciences, Valencia, CA, USA) with buffers supplied by the manufacturer. The full list of genes detected by the SYBR Green-optimized primer assays is shown in (Table 1). A reaction mixture was prepared using 102 μL cDNA, 1248 μL water and 1350 μL SYBR Green/ROX. Analysis was performed using the Sabioscences web portal (http://pcrdataanalysis.sabiosciences.com/pcr/arrayanalysis.php), according to the 2^ΔΔ^CT method. DataSet is assigned a GEO accession number GSE72443.

**Table 1:** The Human Inflammatory Response & Autoimmunity RT² Profiler PCR Array. This assay profiles 84 key genes involved in autoimmune and inflammatory immune responses. It profiles genes related to inflammatory cytokines and chemokines as well as their receptors and also genes related to the metabolism of cytokines and those involved in cytokine-cytokine receptor interactions.

Functional Gene Grouping	Subgroup	Gene symbol
**Cytokine**	**Chemokines**	CCL11 (eotaxin), CCL13 (MCP-4), CCL16 (HCC-4), CCL17 (TARC), CCL19, CCL2 (MCP-1), CCL21 (MIP-2), CCL22 (MDC), CCL23 (MPIF-1), CCL24 (MPIF-2), (Eotaxin-2), CCL3 (MIP-1A), CCL4 (MIP-1B), CCL5 (RANTES), CCL7 (MCP-3), CCL8 (MCP-2), CXCL1 (GRO1, GROa, SCYB1), CXCL10 (INP10), CXCL2 (GRO2, GROb, SCYB2), CXCL3, CXCL5 (ENA-78, LIX), CXCL6 (GCP-2), CXCL9 (MIG).
	**Interleukins**	IL10, IL15, IL17A, IL18, IL1A, IL1B, IL1RN, IL22, IL23A, IL5, IL6, CXCL8
	**Other Cytokines**	IL10, IL15, IL17A, IL18, IL1A, IL1B, IL1RN, IL22, IL23A, IL5, IL6, CXCL8, CSF1(MCSF), FASLG (TNFSF6), LTB, TNFSF14
**Cytokines Receptors**	**Cytokine Receptor**	IL10RB, IL1R1, IL1RAP, IL23R,IL6R.
	**Chemokine Receptors**	CCR1, CCR2, CCR3, CCR4, CCR7, CXCR1 (IL8RA), CXCR2 (IL8RB), CXCR4.
**Cytokine Metabolism**	**-**	IL10, IL18, TLR1, TLR3, TLR4, TLR6
**Cytokine-Mediated Signaling**	**-**	CCL2 (MCP-1), CCL5 (RANTES), CCR1, CCR2, IFNG, IL1A, IL1B, IL1R1, IL1RN, IL5, IL6, IL6R, MYD88, RIPK2, TNF
**Acute-phase response**		CEBP, CRP, PTGS2
**Chronic Inflammatory Response**	**-**	CCL11 (eotaxin), CCL5 (RANTES), IL1B, LTA (TNFB), TNF
**Humoral Immune Response**	**-**	C3, CCL16 (HCC-4), CCL2 (MCP-1), CCL22 (MDC), CCL3 (MIP-1A), CCL7 (MCP-3), CCR2, CCR7, CD40 (TNFRSF5), IL10, IL18, IL1B, IL6, ITGB2, LY96 (MD-2), NFKB1.
**Regulation of the inflammatory response**	**-**	BCL6, C3AR1, CD14, CD40LG, FOS, IL9, KNG91, NOS2, NR3C1, SELE, TIRAP, TLR3, TLR5, TLR7, TOLLIP

#### Quantitative Real-Time PCR

Quantitative PCR (qPCR) was performed to evaluate the possible effects of the *A*. *actinomycetemcomitans* invasion in the lower chamber compartment upon reaching the subepithelial cells (HGF-1). In addition, we aimed to analyze genes related to the inflammation process to verify whether MSE and fractions could promote some biological activity in the infection process. RNA was isolated from cell culture after 24h of treatment using the RNeasy Mini Kit (Qiagen; Valencia, CA, USA). Purity and quantity of RNA were measured in the NanoPhotometer P360 (Implen; Westlake Village, CA, USA). RNA sample has been treated with DNase. Reverse transcription of RNA to cDNA was performed using the QuantiTect Reverse Transcription Kit (Qiagen, Valencia, CA, USA) according to the manufacturer’s instructions. Based on PCR array analysis, genes were selected that presented significant levels of down-regulation (Quantitech Primers, Qiagen). The threshold was manually adjusted within the logarithmic curve above the background level and below the plateau phase. A comparative Ct method was used to calculate the relative gene number. The relative gene copy number was calculated using the 2^ΔΔ^CT method.

#### Cytokine assay

Cytokine assays were performed on all samples using specific enzyme-linked immunosorbent assay (ELISA) kits (Qiagen, Valencia, CA, USA). The cytokines were selected in order to confirm the encoded genes that exhibited down-regulation in the gene expression analyses. The concentration of IL-1alpha, IL-1beta, IL6, IL8, IL10 and GM-CSF were measured according to the manufacturer’s instructions.

## Results

### Chemical analysis

#### Determination of total flavonoid, phenol and condensed tannin content

The phytochemical characterization revealed that the total polyphenol contents of MSE, CLF and AF were 38%, 26% and 22% gallic acid equivalents, respectively. Tannin represented 0.02%, 0.54% and 0.8% catechin equivalent in MSE, CLF and AF respectively; flavonoid content was 2.7%, 7% and 5.6% quercetin equivalent in MSE, CLF and AF respectively.

### Susceptibility testing

Table 2 shows the MIC and the MBC values of MSE and fractions screened for different periodontopathogenic bacteria. The results demonstrated that MSE and CLF had inhibitory activity for the all microorganisms tested: *A*. *actinomycetemcomitans*, *Fusobacterium nucleatum*, *Prevotella intermedia* and *Porphyromonas gingivalis*. CLF was the most potent, with an MIC against *A*. *actinomycetemcomitans* of 150 μg/mL, an MIC against *F*. *nucleatum* of 500 μg/mL and an MIC against *P*. *intermedia* of 125 μg/mL. The MSE had the lowest MIC against *P*. *gingivalis* (15.6 μg/mL). AF had no inhibitory activity against any of the bacteria tested. Gentamicin was used as the positive control (10 μg/mL).

**Table 2:** Minimum inhibitory concentration (MIC) and minimum bactericidal concentration (MBC). MIC and MBC for the ethanolic extract of *Malva sylvestris* and its chloroform and aqueous fraction against four different periodontopathogens: *Aggregatibacter actinomycetemcomitans* D7S1, *Fusobacterium nucleatum* ATCC 25586, *Prevotella intermedia* ATCC 25611 and *Porphyromonas gingivalis* ATCC BAA-308. The highest concentration evaluated was 1000 μg/mL and the minus symbol (-) means no inhibitory activity.

*Extract/fraction*	*A*. *actinomycetemcomitans*		*F*. *nucleatum*		*P*. *intermedia*		*P*. *gingivalis*	
	MIC	MBC	MIC	MBC	MIC	MBC	MIC	MBC
MSE	175	500	1000	-	250	-	15.6	125
CLF	150	250	500	-	125	500	62.5	1000
AF	-	-	-	-	-	-	-	-

### Cell viability test

**Fig 1:**
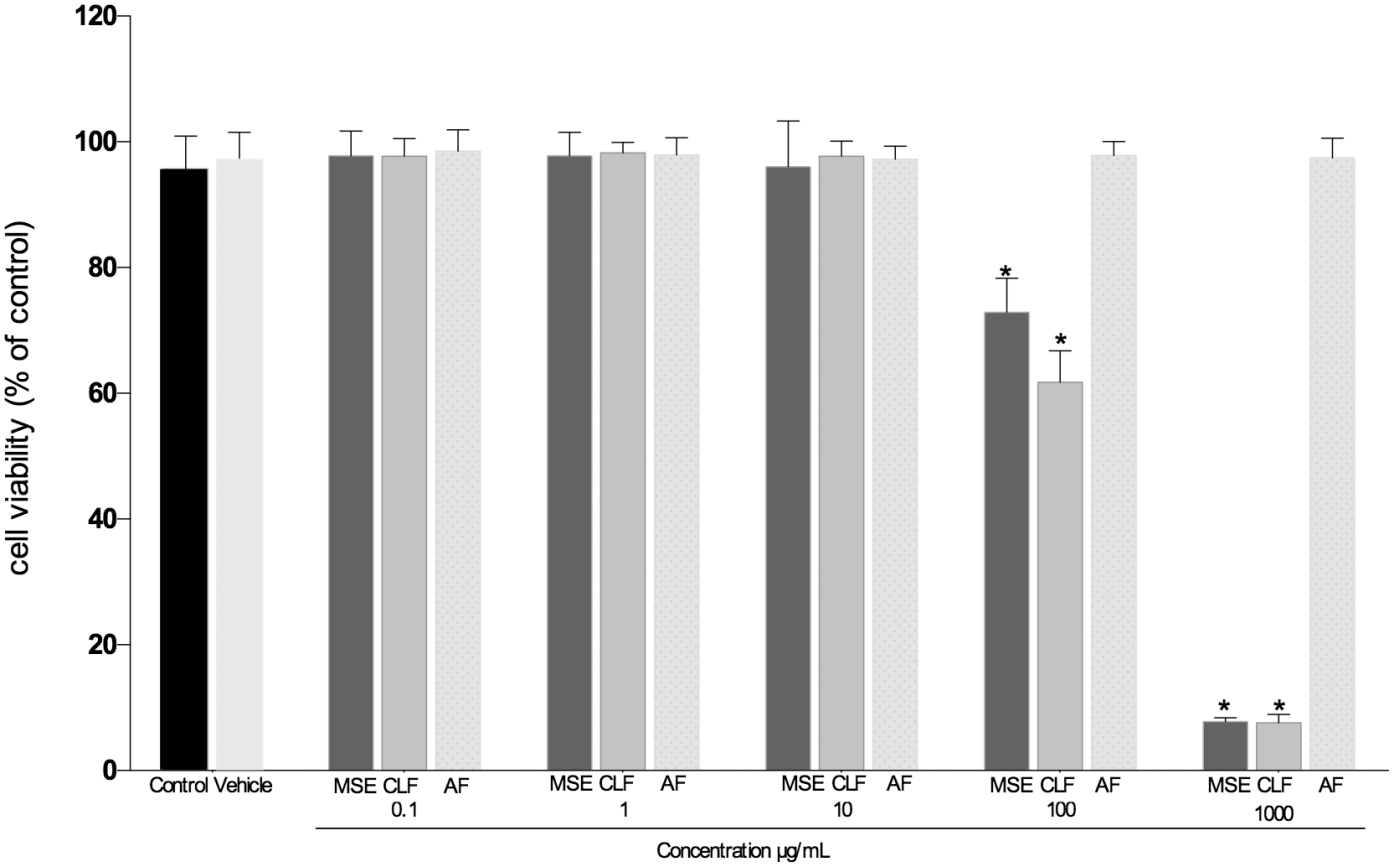
Cytotoxicity test. Cytotoxic effect of MSE, CLF and AF on fibroblasts HGF-1 cells. Data are expressed as mean ± SEM using one-way analysis of variance (ANOVA) followed by Dunnet’s multiple comparison tests as compared to non-treated. The level of statistical significance was set at 0.05.

The cytotoxicity of the extract and all fractions was assessed at concentrations of 0.1, 1, 10, 100 and 1000 μg/mL. AF did not affect the cell viability (p>0.05) at any of the concentrations tested when compared to the control group (non-treated). MSE and CLF reduced the number of viable cells at concentrations of 100 μg/mL and 1000 μg/mL (p<0.05). MSE and CLF were non-toxic at concentrations of 0.1, 1 and 10 μg/mL (Fig 1).

### Invasion dual chamber assay

#### MSE, CLF and AF activity in the co-culture model: susceptibility test, ELISA and gene expression

**Fig 2:**
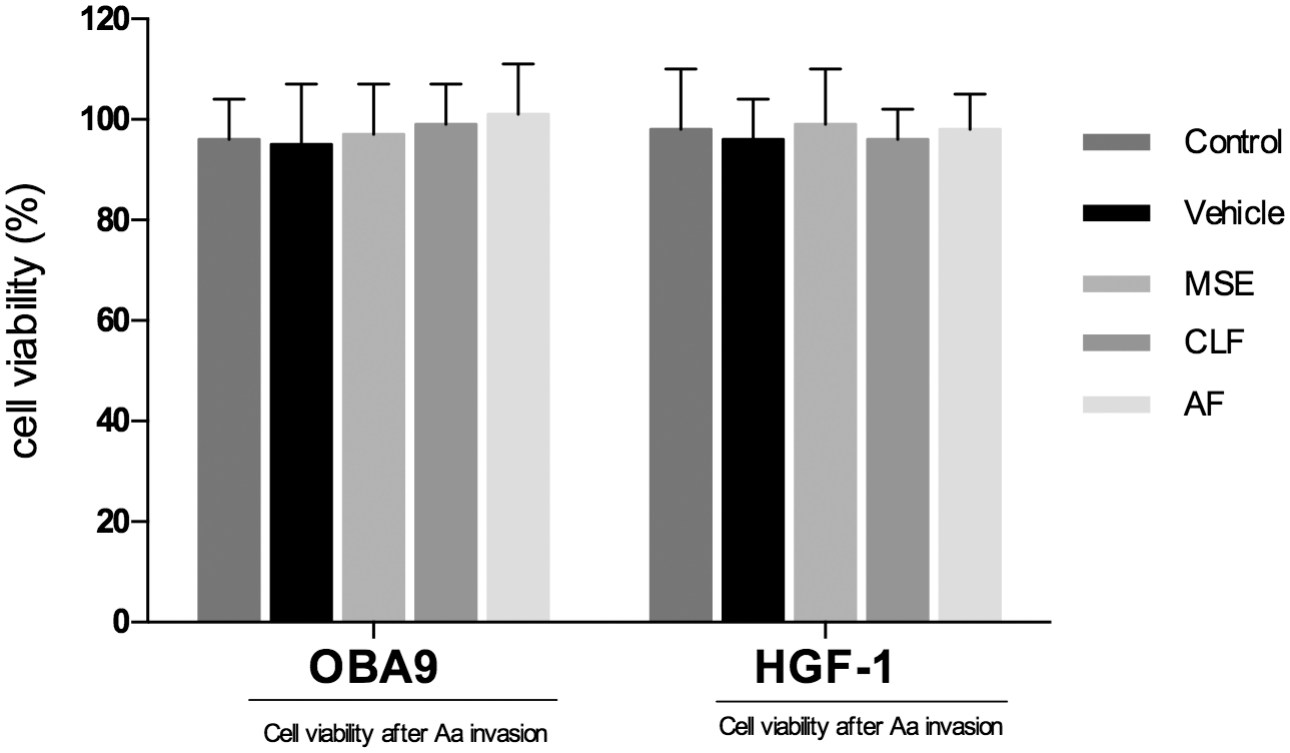
Cytotoxicity effect in the dual chamber model. Cytotoxic effects of MSE, chloroform and aqueous fraction on fibroblast HGF-1 and keratinocyte OBA-9 cell lines were found after 24 h hours of invasion by *A*. *actinomycetemcomitans*. Data are expressed as mean ± SEM using one-way analysis of variance (ANOVA) followed by Dunnet’s multiple comparison tests as compared to the control group (non-treated). The level of statistical significance was set at 0.05.

A viability test was used to determine the effects of the treatments and select the best culture conditions after *A*. *actinomycetemcomitans* infection. In addition, cells were analyzed for the ability to form an impervious epithelial layer by measuring the TEER. Dose-dependent antimicrobial effects were found for MSE and CLF treatment but high concentrations (≥ 100 μg/mL) were toxic for the cells; thus we tested the highest non-toxic concentration for all the samples. A sub-MIC concentration of 75 μg/mL was established. Results confirmed that treatment with 75 μg/mL MSE or CLF did not significantly affect the percentage of viable cells or the integrity of the tight epithelial conjunction (Fig 2).

**Fig 3:**
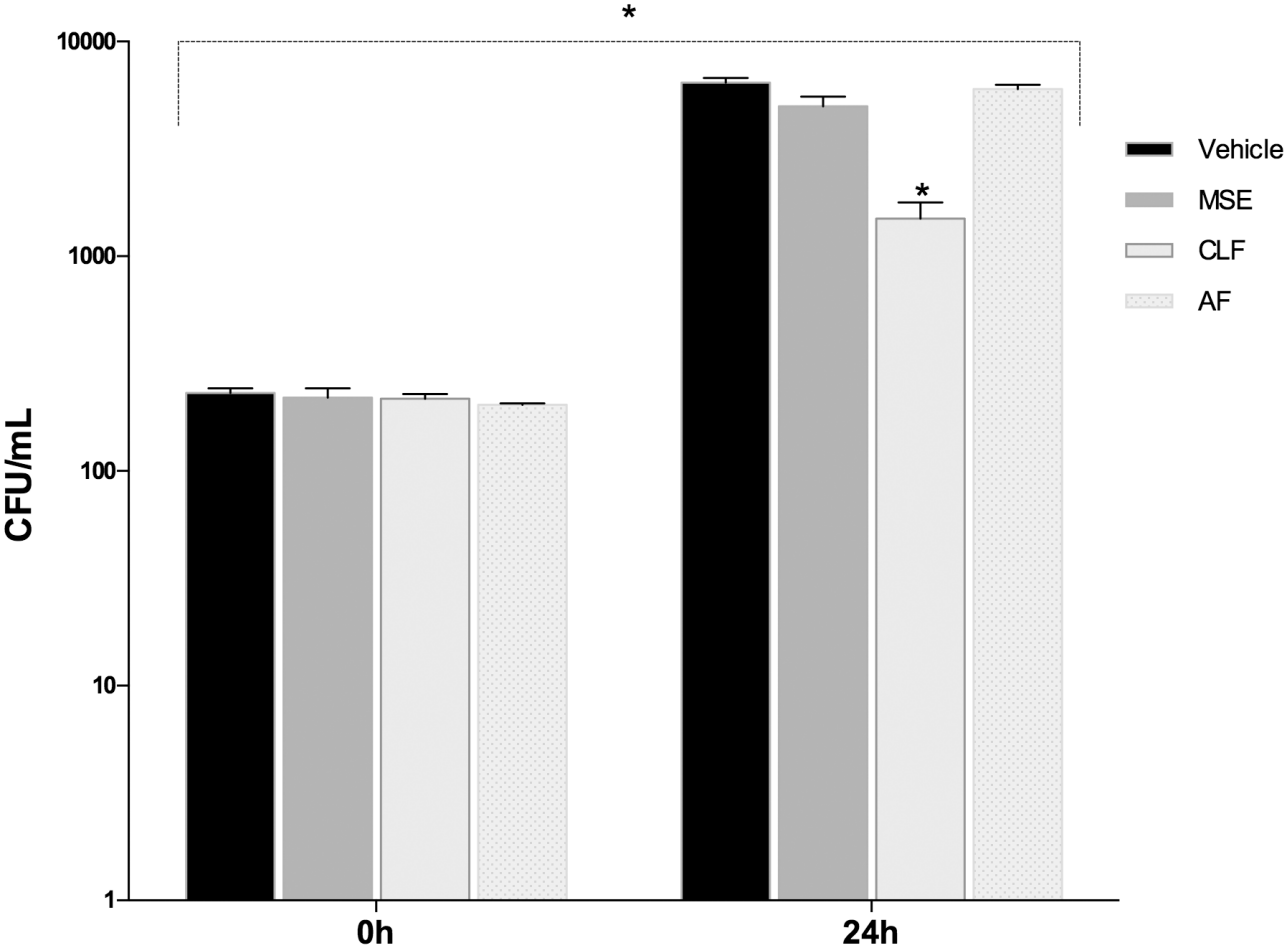
Comparison of colony-forming units. Comparison of colony-forming units (CFU/mL) among groups treated with MSE, CLF and AF after *A*. *actinomycetemcomitans* infection. Data are expressed as mean ± SEM using one-way analysis of variance (ANOVA) followed by Dunnet’s multiple comparison tests as compared to vehicle control. The level of statistical significance was set at 0.05.

In order to confirm the invasion assay of *A*. *actinomycetemcomitans* we first recovered the cultures from the dual compartment chambers. The time 0 h represents this time point at which the CFU quantification was performed after 2h infection period followed by 1h gentamicin treatment. There were no differences among the groups in the amount of internalized bacteria at time 0h (p>0.05). Twenty-four hours after the infection was initiated, the groups treated with MSE and AF had no ability to reduce or eliminate the invasion in the host cells; however, the number of microorganisms in the CLF group was reduced significantly compared to the control vehicle group (p<0.05)(Fig 3).

#### Analysis Using the RT^2^ Profiler PCR Array

Alterations in mRNA transcript levels for all treatment groups were initially analyzed using the RT^2^ Profiler PCR Array and 84 genes were screened and analyzed using the SABiosciences web portal software. The transcriptional profile in the lower chamber cell lines after 24 hours invasion assay is showed on Table 3. It was found a down-regulation of 6 different genes among 84 target genes. For the fold changes, values less than 1 are considered down regulated.

**Table 3:** The Human Inflammatory Response & Autoimmunity RT² Profiler PCR Array. Genes in the inflammatory pathway down-regulated by the treatments with MSE, CLF and AF.

Symbol	Gene	Gen Bank	Fold changes		
			MSE	CLF	AF
BCL6	B-cell CLL/lymphoma 6	NM_001130845	0.37	0.45	0.68
CD14	CD14 molecule	NM_000591	0.64	0.63	0.57
FOS	FBJ murine osteosarcoma viral oncogene homolo**g**	NM_005252	0.23	0.05	0.81
IL-1beta	Interleukin 1, beta	NM_000576	0.57	0.64	0.57
IL-6R	interleukin 6 receptor	NM_000565	0.07	0.08	0.29
IL-8	Interleukin 8	NM_000584	0.36	0.48	0.89

#### Quantitative Real-Time PCR

The following genes were analyzed using qRT-PCR: IL-1alpha, IL-1beta, IL-6, IL-8, IL-10, CD14, PTGS, FOS, BCL6 and MMP-1. The reference control gene was GAPDH and the calibrator for the 2^ΔΔ^CT method was the non-treated control group. A minus-reverse transcriptase control was included in all qPCR experiments.

**Fig 4:**
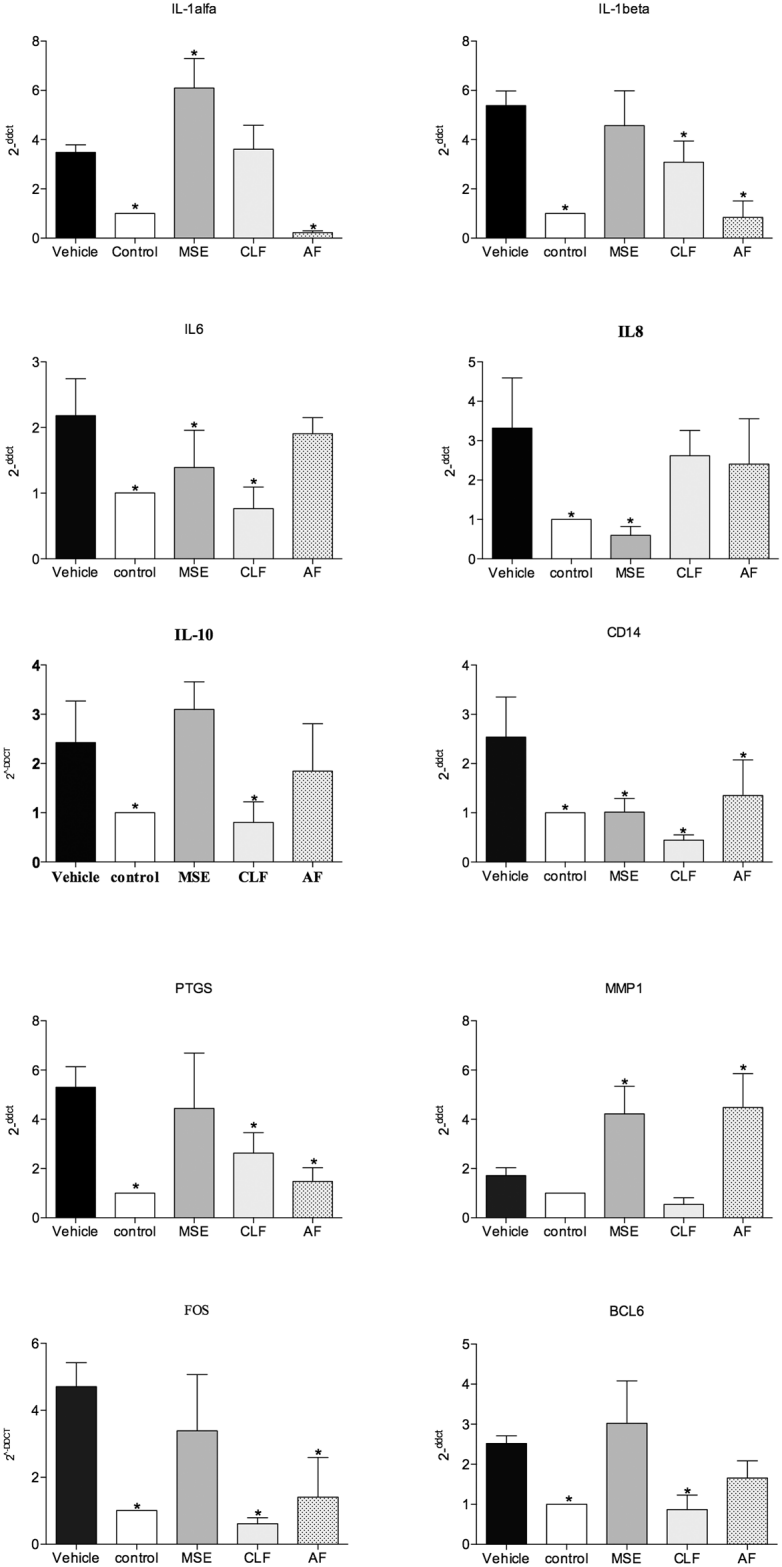
Gene expression analysis of lower chamber co-culture invasion assay. The expression levels of IL-1alfa, IL-1beta, IL-6, IL-8, IL-19, CD14, PTGS, MMP-1, FOS, BCL2 were evaluated and compared to the control (non-infected cells). Quantification of the relative transcript amounts was performed using qPCR with 50 ng of each cDNA. Data quantification was performed using the 2^ΔΔ^CT method. Statistical analyses included one-way ANOVA followed by Dunnet’s post-hoc tests. A significance level of p < 0.05 (*) indicates differences from the vehicle group.

The results showed that cells invaded by *A*. *actinomycetemcomitans* and treated with MSE had statistically significant down-regulation of the genes IL6, IL8, CD14 compared to the control group (non-infected cells) (p<0.05). The chloroform fraction down-regulated the gene expression of IL-1beta, IL6, IL10, PTGS, CD14, PTGS, FOS and BCL6 (p<0.05) compared to the control group. The group treated with AF showed a down-regulation of mRNA transcript levels for the genes IL-1alpha, IL-1beta, CD14, PTGS and FOS (p<0.05) (Fig 4).

#### Cytokine assay

**Fig 5:**
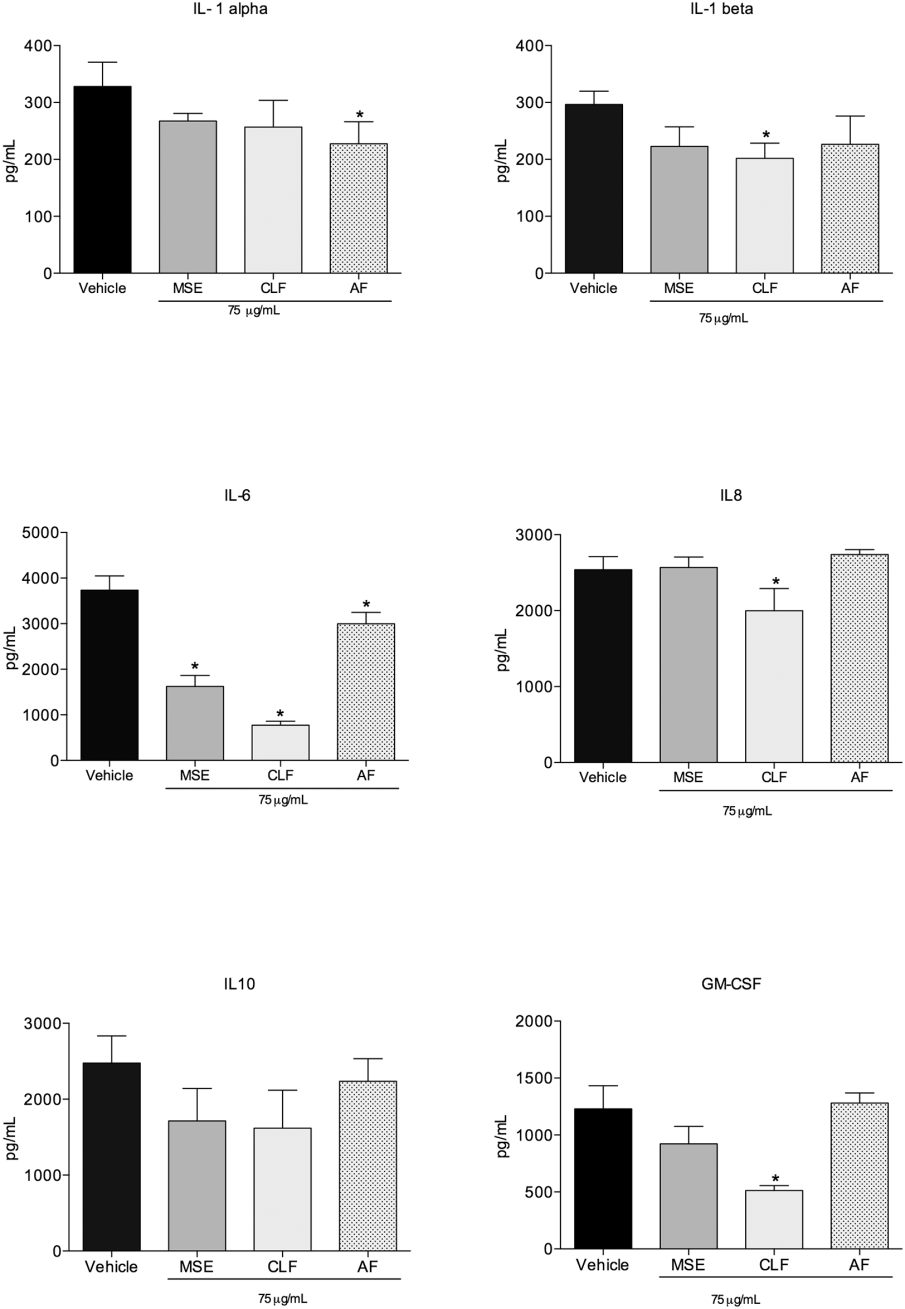
Cytokine assay. Quantification of IL-1alpha, IL-1beta, IL-6, IL-8, IL-10 and GM-CSF in the co-culture supernant after 24h of *A*. *actinomycetemcomitans* invasion. Cells were treated with 75μg/mL of MSE and fractions and bacteria inocula were established at 2x10^6^ CFU/mL. Data are expressed as mean±SD, n = 6. Symbols indicate statistical differences (p<0.05, Dunnet’s test). # indicates p<0.05 compared to non-treated group; * indicates p<0.05 compared to vehicle group.

The concentrations of IL-1 alpha, IL-1beta, IL-6, IL-8, IL-10 and GM-CSF were quantified by ELISA to confirm whether the proteins encoded by the down-regulated genes were found at reduced levels in the supernatant. MSE reduced the expression of IL-6 in the infected cells compared to the vehicle control group (p<0.05). Significant differences in the levels of a number of cytokines were found between the CLF and control groups. Specifically, reduced expression levels were observed for the cytokines IL-1beta, IL-6, IL-8 and GM-CSF (reductions of 32%, 80, 3%, 30, 58 and 4% compared to the vehicle control, respectively). Infected cells expressed low levels of IL-1 alpha when treated with the AF, a decrease of 31% compared to the vehicle control group (Fig 5).

## Discussion

Periodontal disease is an oral infectious inflammatory disease and the most common human chronic disorder [23]. The relationship between this disease and many systemic diseases (including cardiovascular disease, diabetes, adverse pregnancy outcomes and others) is well recognized [24, 25]. Based on current knowledge of inflammation pathways, it appears that natural products may be a good source for developing multi-target drugs with activity against the microorganisms responsible for periodontal disease [26, 27].

For this study, we evaluated the toxicity, antimicrobial and anti-inflammatory activity of compounds naturally occurring in the plant *M*. *sylvestris* [28]. A screening assay simulating the effect of *A*. *actinomycetemcomitans*, a species known to be associated with periodontal disease, was used to model the infection of epithelial and subepithelial cell lines [22]. These results confirmed the internalization of the bacteria, indicating the possible activation of the membrane and intracellular receptors [29]. Transcriptional factors and cytokines identified in the infection process suggested signaling and host response pathways were involved in the bacteria challenge and during the treatment with *M*. *sylvestris* extract and fractions.

The antimicrobial susceptibility test showed that MSE and CLF had activity not only against *A*. *actinomycetemcomitans* but also against other periodontopathogens (*F*. *nucleatum*, *P*. *gingivalis* and *P*. *intermedia*) that are implicated in the development and virulence of periodontal disease [1]. In addition, this demonstrates that *M*. *sylvestris* works against both microaerophiles and anaerobes. In the literature, it has been shown that the ethanolic extract of *M*. *sylvestris* is effective as a bacteriostatic agent against methicillin-resistant *S*. *aureus* (I_50_ ≤ 32 μg/ml) [30], and moderate to low activity was reported against strains of *Helicobacter pylori* (MIC ranged from 0.625 to >5.0 mg/mL)[31]; moreover, the aqueous fraction was reported to have anti-fungal activity, though not against *Candida albicans* [32]. Overall, though antimicrobial effects of *M*. *sylvestris* have been reported in the literature for a few microorganisms [30, 31, 32] these studies used different extract preparations and the majority were based on agar-diffusion tests, making inter-study comparisons difficult.

Our findings also demonstrate that the bioguided fractionation was successful and may be a model for bioprospecting new drugs, as long as the active fraction (CLF) presented enhanced antimicrobial activity relative to the unfractionated extract. In addition, our data describe the cytotoxicity of the extract and fractions *in vitro* to provide better estimation of the potential of the compound as favorable therapeutic agent. The viability test showed that the CLF fraction was non-toxic at concentrations up to 100 μg/mL and AF had no toxic effects at any of the concentrations tested. The LD_50_ of the extract for cell lines OBA-9 and HGF (250 μg/mL and 210 μg/mL, respectively) gave insight into the safe concentrations for use in the biological assays. *M*. *sylvestris* is widely known as a food or condiment and has been used for millennia in traditional medicine; however, only one *in vivo* test of its toxicity has been reported in the literature [33].

The bacterial products from *A*. *actinomycetemcomitans* affected the cell immune response and increased the production of local cytokines. All the treatments tested affected different signaling pathways. Upon treatment with the aqueous fraction, both the IL-1alpha gene and protein expression levels were reduced. The pro-inflammatory cytokine IL-1 and tumor necrosis factor alpha (TNF alpha) are modulators of the host response to microbial infection. It has previously [34] been demonstrated that IL-1 specific marker is a strong indicator of susceptibility to severe periodontal disease in adults. Furthermore, it has been established that IL-1 is involved in the induction of bone resorption by promoting the differentiation of osteoclast precursors in active osteoclasts [35].

A statistical reduction of IL-6 gene expression and protein levels were found after treatment with the chloroform fraction (CLF). The higher expression levels of IL-6 in untreated periodontal disease might induce an increase in matrix metalloproteinases (MMPs) that are related to tissue destruction [36, 37]. IL-6 has been reported as a principal regulator in the acute phase of inflammation and may promote osteoclastogenesis by increasing tRANKL expression [38].

In addition, the CLF treatment regulated the expression of other immunomodulatory genes (CD14, MMP1 and FOS), which indicates an effect on more than one signaling pathway and may result in a good therapeutic outcome. Finding compounds that trigger CD14 or toll-like receptors (TLRs) is potentially useful in periodontal disease. The binding of lipopolysaccharides (LPS) with CD14 might induce the temporary activation of many protein kinases and the phosphorylation of intracellular proteins essential for LPS activation in monocytes/macrophages [39].

The MSE could regulate the transcription of IL-8 but not the same cytokine expression. The answer to the question of how genomic information can be processed differently to produce a specific cellular proteome to date remains unanswered [40, 41]. The literature has been demonstrated that *M*. *sylvestris* may regulated the expression of cytokines in the inflammatory process. In a pre-clinical study, important anti-inflammatory action of the hydroalcoholic extract was found to interfere with the production of IL-1beta and consequently block leukocyte migration [42]. Furthermore, the aqueous extract of *M*. *sylvestris* was found to have an immunomodulatory property, acting as a macrophage activators and promoting both IL-12 and (IFN) interferon transcripts [42]. Overall, the literature and present data highlight the biological activity of *M*. *sylvestris* in treating inflammation.

The phytochemical investigation of *M*. *sylvestris* showed a high occurrence of phenolic compounds in all studied extracts and fractions. This is consistent with a previous report [14], in which 4-hydroxybenzoic acid, 4-methoxybenzoic acid, 4-hydrocycinnamic acid and tyrosol were isolated from *M*. *sylvestris*. Furthermore, the interest in phenolic compounds has increased in recent years due to their possible implications for human heath, such as in treating and preventing cancer, cardiovascular disease and other pathologies [11]. Overall, phenolic compounds are particularly potent natural products with a wide range of biological properties known in the literature that could be used extensively in dentistry.

The results of the present study showed that the low-polarity fraction CLF has relevant dual activity, simultaneously controlling infection and inflammation processes. Thus, *M*. *sylvestris* may be considered as a potential drug candidate for use as a new therapeutic approach in the treatment of the periodontal disease.

## Conclusion

In our study we found that *Malva sylvestris* and its chloroform fraction were able to minimize the infection and inflammation process in oral human cells by a putative pathway that may involve the antimicrobial effect and modulation of cytokines and receptors. Therefore, this natural product may be considered as a successful dual anti-inflammatory–antimicrobial candidate.
